# Tetrandrine Interaction with ABCB1 Reverses Multidrug Resistance in Cancer Cells Through Competition with Anti-Cancer Drugs Followed by Downregulation of ABCB1 Expression

**DOI:** 10.3390/molecules24234383

**Published:** 2019-11-30

**Authors:** Dan Liao, Wei Zhang, Pranav Gupta, Zi-Ning Lei, Jing-Quan Wang, Chao-Yun Cai, Albert A. De Vera, Lei Zhang, Zhe-Sheng Chen, Dong-Hua Yang

**Affiliations:** 1 Key Laboratory for Complementary and Alternative Medicine Experimental Animal Models of Guangxi, Guangxi University of Chinese Medicine, Nanning 530200, China; dan9999@126.com; 2 Department of Pharmaceutical Sciences, College of Pharmacy and Health Sciences, St. John’s University, Queens, NY 11439, USA; zhangwei@wfmc.edu.cn (W.Z.); zining.lei14@my.stjohns.edu (Z.-N.L.); jingquan.wang16@my.stjohns.edu (J.-Q.W.); chaoyun.cai16@my.stjohns.edu (C.-Y.C.); albert.devera13@my.stjohns.edu (A.A.D.V.); zhangl@fjirsm.ac.cn (L.Z.); 3 Institute of Plastic Surgery, Weifang Medical University, Weifang 261041, China; 4 State Key Laboratory of Structural Chemistry, Fujian Institute of Research on the Structure of Matter, Chinese Academy of Sciences, Fuzhou 350002, China

**Keywords:** ABC transporter, tetrandrine, multidrug resistance (MDR), ABCB1/P-gp, transgenic cancer cell

## Abstract

The overexpression of ABC transporters induced by anticancer drugs has been found to be the main cause of multidrug resistance. It is actually also a strategy by which cancer cells escape being killed. Tetrandrine is a natural product extracted from the stem of *Tinospora crispa*. In this study, tetrandrine showed synergistic cytotoxic activity in combinational use with chemotherapeutic drugs, such as Doxorubicin, Vincristine, and Paclitaxel, in both drug-induced and *MDR1* gene-transfected cancer cells that over-expressed ABCB1/P-glycoprotein. Tetrandrine stimulated P-glycoprotein ATPase activity, decreased the efflux of [^3^H]-Paclitaxel and increased the intracellular accumulation of [^3^H]-Paclitaxel in KB-C2 cells. Furthermore, SW620/Ad300 and KB-C2 cells pretreated with 1 μM tetrandrine for 72 h decreased P-glycoprotein expression without changing its cellular localization. This was demonstrated through Western blotting and immunofluorescence analysis. Interestingly, down-regulation of P-glycoprotein expression was not correlated with gene transcription, as the *MDR1* mRNA level exhibited a slight fluctuation in SW620/Ad300 and KB-C2 cells at 0, 24, 48, and 72 h treatment time points. In addition, molecular docking analysis predicted that tetrandrine had inhibitory potential with the ABCB1 transporter. Our results suggested that tetrandrine can antagonize MDR in both drug-selected and *MDR1* gene-transfected cancer cells by down regulating the expression of the ABCB1 transporter, followed by increasing the intracellular concentration of chemotherapeutic agents. The combinational therapy using tetrandrine and other anticancer drugs could promote the treatment efficiency of drugs that are substrates of ABCB1.

## 1. Introduction

The ATP-binding cassette (ABC) transporters are cell membrane ATP-dependent efflux pumps that transport substrates from intracellular to extracellular space. They are a superfamily of protein transporters. In humans, 49 members have been found and sub-divided into seven subfamilies with names ranging from ABCA to ABCG [1]. Previous studies have demonstrated that the main mechanism of cancer multidrug resistance (MDR) is closely associated with over-expression of these trans-membrane proteins. These proteins can directly decrease the intracellular drug concentration [2]. ABCB1, also named P-glycoprotein (P-gp/MDR1), was the first one identified and confirmed as a human mammalian ABC transporter [3]. This membrane protein has been found to be extensively expressed in many normal tissues [4], such as the placenta, kidney, liver, intestine, adrenal gland, lymphocytes, heart small blood vessels, and blood-brain barrier. The molecular weight of ABCB1 is 170 Kd and it comprises of two homological parts, where each contains a six-transmembrane α-helices domain (TMD) and a cytosolic nucleotide-binding domain (NBD). The TMD combines and transports substrates, and the NBD has an ATP binding site and possess ATPase activity to provide energy from hydrolysis of ATP [5]. Overexpression of ABCB1 is bound to promote the efflux of diverse anticancer drugs and results in MDR. Overexpression of ABCB1 was found in various carcinomas such as non-small cell lung cancer [6], breast cancer [7], gastric cancer [8], nasal NK/T cell lymphoma [9], T/NK-cell lymphomas [10], prostate cancer [11], ovarian carcinoma [12], and leukemia [13]. In clinics, these carcinomas showed resistance to related chemotherapeutic drugs such as taxanes, vinca alkaloids, epipodophyllotoxins, anthracyclines, and tyrosine kinase inhibitors, and these drugs are known as substrates of ABCB1 [14]. Incapacitation of anticancer drugs can be triggered by the overexpression of ABCB1, making the therapy for cancer difficult or even fail. In order to overcome this predicament, one strategy is to combine anticancer drugs with chemosensitizer simultaneously. Previous research revealed that there were some active compounds exist in nature that could reverse multidrug resistance by reducing the function of ABCB1 protein.

Tetrandrine, a natural compound extracted from traditional Chinese herb *Tinospora crispa*, has been reported to possess remarkable antitumor activity in many types of cancers both in vitro and in vivo in the past 20 years. The main mechanisms of action of tetrandrine were related to multiple factors such as modulating molecular signaling pathways [15], inducing cancer cells apoptosis [16], promoting cell cycle arrest [17], and increasing cell autophagy [18]. Previous studies show that tetrandrine could reverse MDR mediated by ABCB1 transporter in acquired resistant cancer cells such as MCF-7/Adriamycin [19], KBv200/vincristine [20], HCT15 (ABCB1 positive) [21] , multidrug resistance T lymphoblastoid leukemia cell line MoLT-4 [22], hepatocellular carcinoma Bel-7402/Adriamycin [23], mouse leukemia cell P388/Adriamycin [24], K562/doxorubicin [25], and T-cell acute lymphoblastic CEM/Adr5000 [26], osteosarcoma cell line U-2os [15], other multidrug resistant OS cell lines [27], and the human gastric carcinoma cell line SGC7901 [28]. However, there has not been any study on MDR mediated by ABCB1 transporter in gene transfected cells. In this study, we explore whether the reversal effect of tetrandrine on multidrug resistance is the same in drug selected and *MDR1* gene transfection cells. Three pairs of cell lines KB-3-1 and KB-C2, SW620 and SW620/Ad300, and HEK293/pcDNA3.1 and HEK293/ABCB1 were used to investigate whether tetrandrine could serve as a chemosensitizer.

## 2. Results

### 2.1. Cytotoxicity of Tetrandrine in Both Sensitive and Resistant Cancer Cells

**Figure 1:**
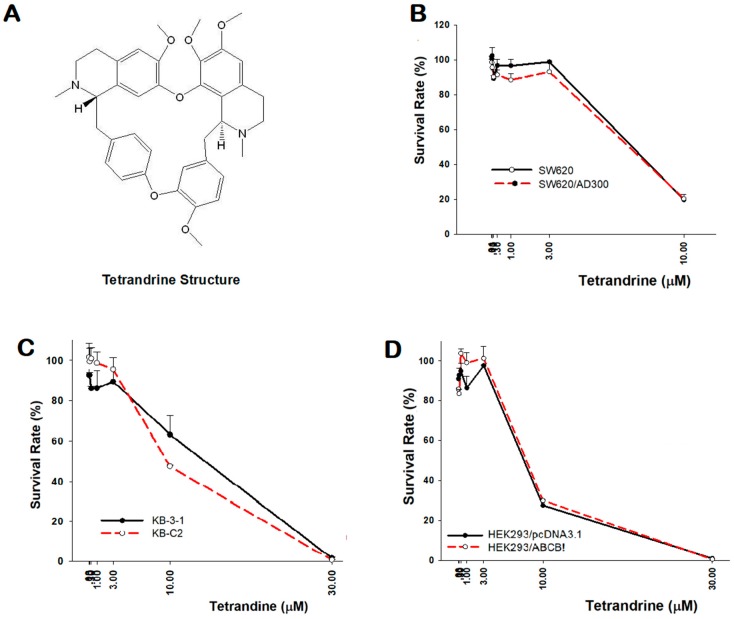
Cytotoxicity of tetrandrine in the parental and resistant cell lines. (**A**) Chemical structure of tetrandrine. MTT assay on the effect of tetrandrine in cells: (**B**) SW620 and SW620/Ad300; (**C**) KB-3-1 and KB-C2; (**D**) HEK293/pcDNA3.1 and HEK293/ABCB1.

Before the reversal experiments, cytotoxicity of tetrandrine was tested in both parental and resistant cancer cell lines using the MTT method, which is an assay used to assess cell viability. The results showed that tetrandrine has a similar effect on reducing cell proliferation in several pairs of sensitive and resistant cell lines: SW620 and SW620/Ad300, KB-3-1 and KB-C2, HEK293/pcDNA3.1 and HEK293/ABCB1. In addition, their IC_50_ values were found to be approximately the same (Table 1, Figure 1).

### 2.2. Reversal Effect of Tetrandrine in ABCB1 Overexpressing Cancer Cells

**Figure 2:**
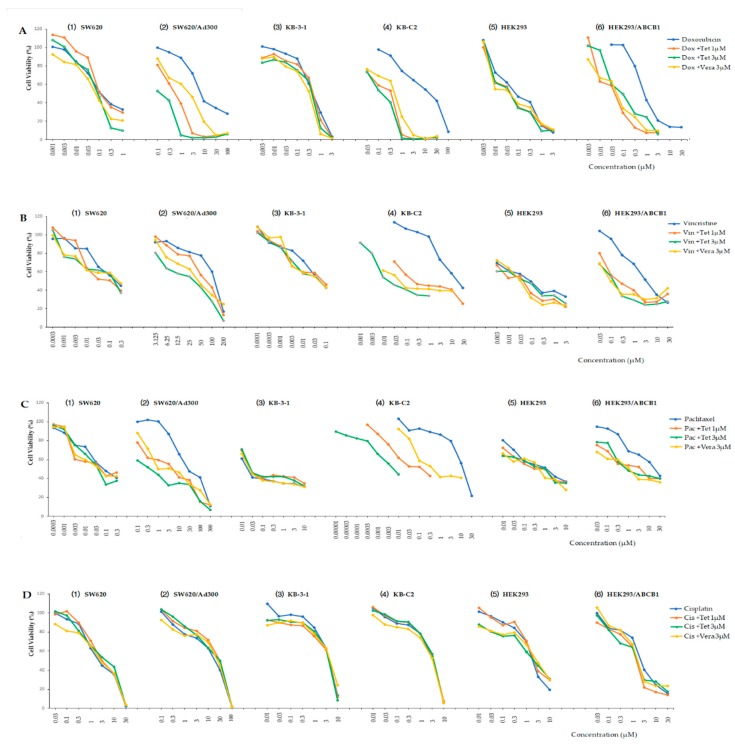
Cell viability assay in three pairs of cells treated with different chemical substrates singly or in combination with 1 μM or 3 μM tetrandrine. **A**(**1**)–(**6**): Doxorubicin with and without 1 μM or 3 μM tetrandrine (Dox + Tet); **B**(**1**)–(**6**): Vincristine with and without 1 μM or 3 μM tetrandrine (Vin + Tet); **C**(**1**)–(**6**): Paclitaxel with and without 1 μM or 3 μM tetrandrine (Pac + Tet); **D**(**1**)–(**6**): control Cisplatin with or without 1 μM or 3 μM tetrandrine (Cis + Tet).

**Figure 3:**
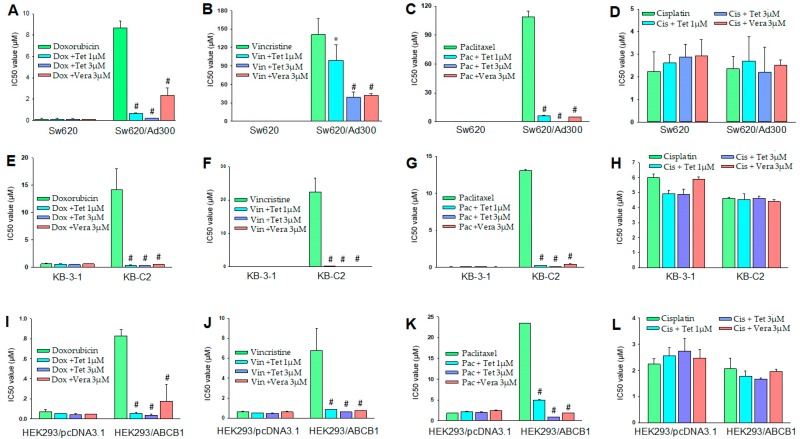
Tetrandrine combined with different anti-cancer drugs reverses the ABCB1-mediated drug resistance in ABCB1 overexpressing cell lines. (**A**–**D**): Sw620 and Sw620/Ad300; (**E**–**H**): KB-3-1 and KB-C2; (**I**–**L**): HEK293/pcDNA3.1 and HEK293/ABCB1. * *p* < 0.05, ^#^ *p* < 0.01 versus the no tetrandrine group.

In order to investigate whether tetrandrine can inhibit the multidrug resistance by affecting the ABC transporter in cell membrane, a non-toxic concentration of tetrandrine was used and combined with different chemical substrates of ABCB1 transporter in these three pairs of parental and resistant cells. Figure 2 showed the cell viability of these cells treated with three different chemical substrates with and without 1 μM or 3 μM tetrandrine, and their IC_50_ values were compared. Based on the cytotoxicity assay mentioned above, 1 μM and 3 μM were considered as non-toxic concentrations of tetrandrine (Figure 1). Our data showed that tetrandrine significantly enhanced the sensitivity of chemical drugs in drug resistant cancer cell lines SW620/Ad300, KB-C2, and HEK293/ABCB1 that overexpressed ABCB1 in a dose-dependence manner (Figure 3, Table 2). Its reversal effect was better than that of verapamil, which was used as a positive control, when tetrandrine was used in combination with Doxorubicin and Paclitaxel. The reversal effect of 1 μM and 3 μM tetrandrine plus Doxorubicin in SW620/Ad300 cells was close to that of using Doxorubicin alone with the sensitive cell SW620. The effect of 1 μM and 3 μM tetrandrine plus Doxorubicin in KB-C2 and HEK293/ABCB1 cells was better than that of using Doxorubicin alone with sensitive cell lines KB-3-1 and HEK293/pcDNA3.1. Similar results were obtained in the combinational use of tetrandrine with Vincristine in KB-C2 cells, and the combinational use of Paclitaxel in HEK293/ABCB1 cells.

### 2.3. Tetrandrine Prevents Efflux and Increases Intracellular Accumulation of [^3^H]-Paclitaxel in ABCB1 Overexpressing Cells

**Figure 4:**
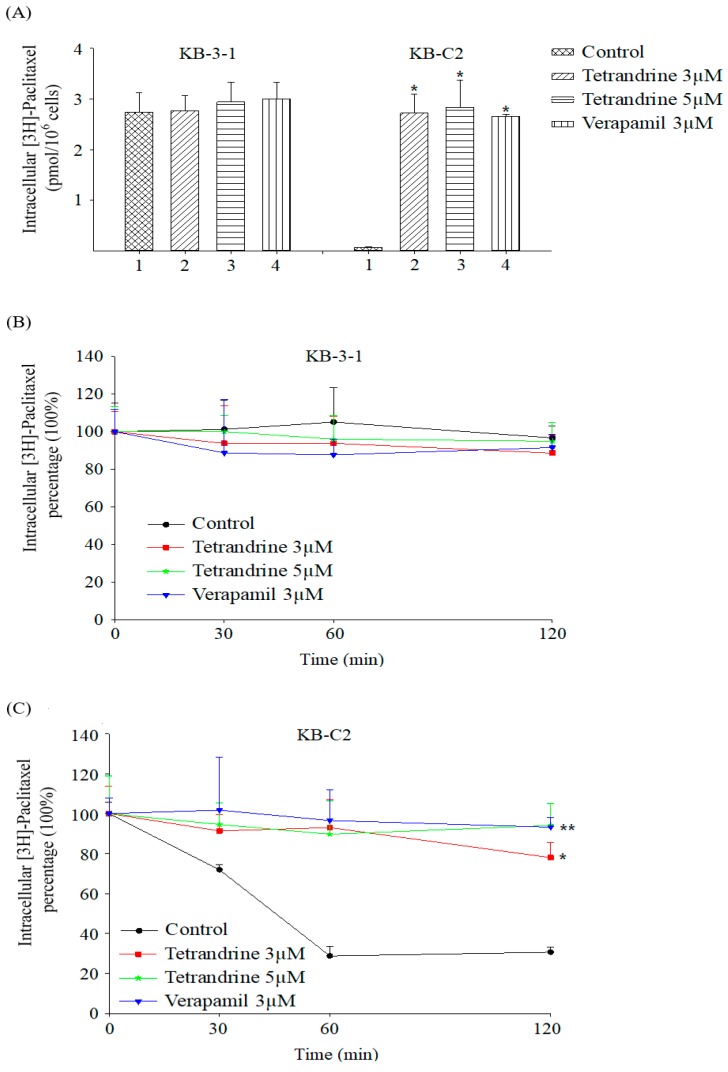
(**A**) Intracellular [^3^H]-paclitaxel in parental cells KB-3-1 and ABCB1-overexpressing cells KB-C2 pretreated with or without tetrandrine (3 μM and 5 μM) and Verapamil (3 μM). (**B**) Intracellular [^3^H]-paclitaxel in parental KB-3-1 cells at different time points. (**C**) Intracellular [^3^H]-paclitaxel in ABCB1-overexpression KB-C2 cells at different time points. Verapamil, an inhibitor of ABCB1, was used as a positive control. * *p* < 0.05, versus the controls.

An important mechanism of MDR was that the ABCB1 transporter in cell membrane pumps out substrates that have been taken into the cells. In order to investigate how tetrandrine reverses MDR, [^3^H]-Paclitaxel, a substrate of ABCB1 transporter, was used to examine the dynamic drug concentration in parental KB-3-1 and resistant KB-C2 cells. It was found that the intracellular accumulation of [^3^H]-paclitaxel in the absence of potential reversal agent tetrandrine was obviously lower than that in the presence of 3 μM and 5 μM tetrandrine in ABCB1-overexpession cells. Verapamil (3 μM) was used as a positive control inhibitor of ABCB1, and its reversal effect was almost the same as that of tetrandrine at concentrations of 3 μM or 5 μM. In comparison, intracellular accumulation of [^3^H]-paclitaxel with or without reversal agent in parental KB-3-1 were similar (Figure 4A). Furthermore, intracellular [^3^H]-paclitaxel was detected in 0, 30, 60, and 120 min time points and the data showed that tetrandrine might maintain [^3^H]-paclitaxel in ABCB1-overexpression cells. As shown in Figure 4B, intracellular [^3^H]-paclitaxel were stable in KB-3-1 parental cells, while intracellular residual [^3^H]-paclitaxel had lost more than 70% in the first 1 h in the absence of tetrandrine (Figure 4C).

### 2.4. Tetrandrine Stimulates the ATPase Activity of ABCB1

**Figure 5:**
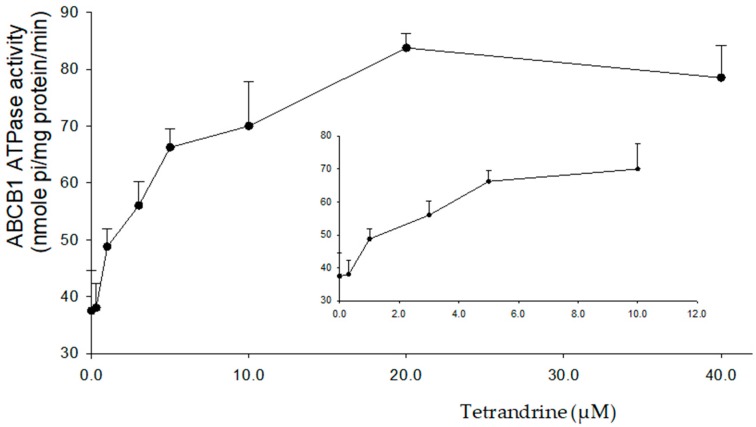
Variation of orthovanadate sensitive ABCB1 ATPase activity with increasing concentration of tetrandrine from 0 to 40 μM.

The amount of free phosphate group (Pi) produced from ATP hydrolysis indirectly reflects the ATPase activity. The change of ATPase activity upon the treatment of tetrandrine was tested. It was found that tetrandrine stimulates the ABCB1 ATPase activity in a concentration-dependent manner from 0 to 40 µM. The maximum stimulation was 2.23-fold higher than that of the basal ATPase activity. In order to get 50% maximum stimulation, 0.47 µM of tetrandrine was required (Figure 5).

### 2.5. Tetrandrine Reduces ABCB1 Protein Expression without Changing its Cellular Localization

**Figure 6:**
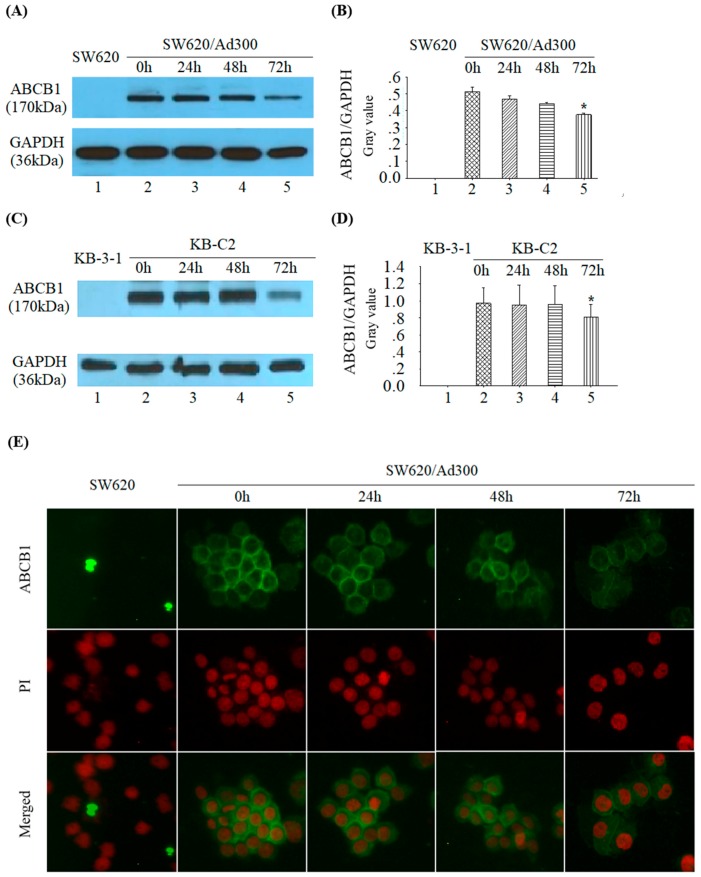
(**A**) The expression of ABCB1 and GAPDH in SW620/Ad300 cells after treatment with 1 µM tetrandrine at 0, 24, 48, and 72 h time points. (**B**) representation of ABCB1/GAPDH relative ratio of A; (**C**) The expression of ABCB1 and GAPDH in KB-C2 cells after treatment with 1 µM tetrandrine at 0, 24, 48, and 72 h time points. (**D**) representation of ABCB1/GAPDH relative ratio of C; (**E**) Immunofluorescent staining on the influence of tetrandrine on the subcellular localization of ABCB1 in SW620/Ad300 cells at 0, 24, 48, and 72 h time points. The nuclei were stained by PI (Propidium Iodide) and shown in red.

The ABCB1 protein expression in SW620/Ad300 cells was detected by Western blotting assay. Target proteins of 170 kDa ABCB1 and internal indicator 36 kDa GAPDH were showed in Figure 6A. ABCB1 expression reduced after pretreatment with tetrandrine at 1 μM for 72 h. Parental cells SW620 with no ABCB1 expression was used as a control. The ratio of ABCB1/GAPDH in SW620/Ad300 significantly decreased at 72 h time point (Figure 6A,B). Down regulation of ABCB1 was also observed in KB-C2 cells (Figure 6C,D). In order to know whether tetrandrine affects the cellular localization of ABCB1, immunofluorescence staining was performed and it showed that the localization of ABCB1 transporter did not change in the cell membrane of SW620/Ad300 cells after incubation with tetrandrine at 1 μM for 0, 24, 48, and 72 h (Figure 6E). These results indicated that tetrandrine reduced the expression of ABCB1 transporter without changing its cellular localization. There was no detectable ABCB1 expression in parental SW620 cells and KB-3-1 cells (Figure 6A,C).

### 2.6. Tetrandrine Does Not Alter the mRNA Expression of ABCB1

**Figure 7:**
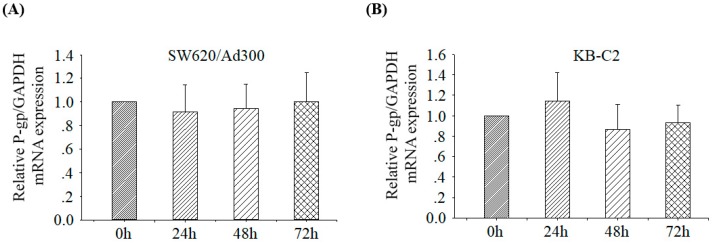
Relative ABCB1/GAPDH mRNA expression of gene *MDR1* in MDR cells after treatment with 1 μM tetrandrine for 0, 24, 48, or 72 h. (**A**) SW620/Ad300 cells; (**B**) KB-C2 cells. The difference was not statistically significant (*p* > 0.05).

Real time PCR test was conducted to analyze the effect of tetrandrine on the mRNA expression of ABCB1 in two cell lines SW620/Ad300 and KB-C2. The results were showed in Figure 7. It was found that the expression of ABCB1 mRNA was not significantly affected after treatment with 1 μM tetrandrine for 0, 24, 48, and 72 h. This result suggested that the downregulation of protein expression of ABCB1 was not at the transcriptional level after treatment with tetrandrine.

### 2.7. Molecular Modeling Analysis

**Figure 8:**
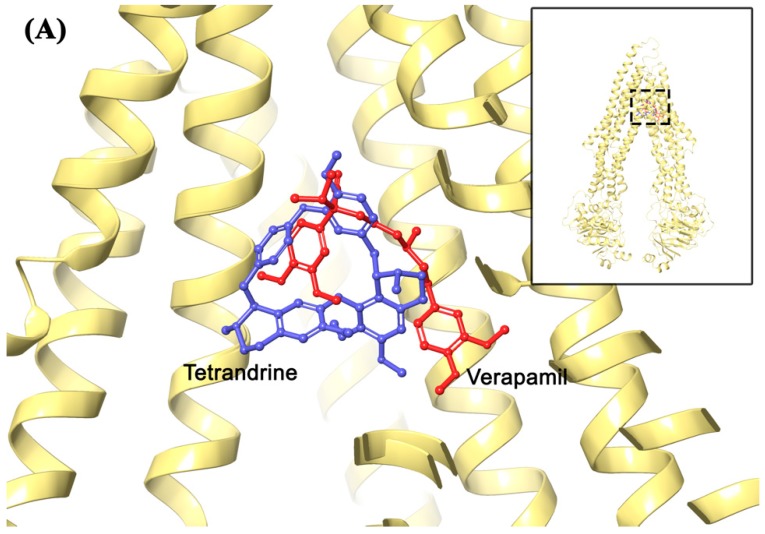

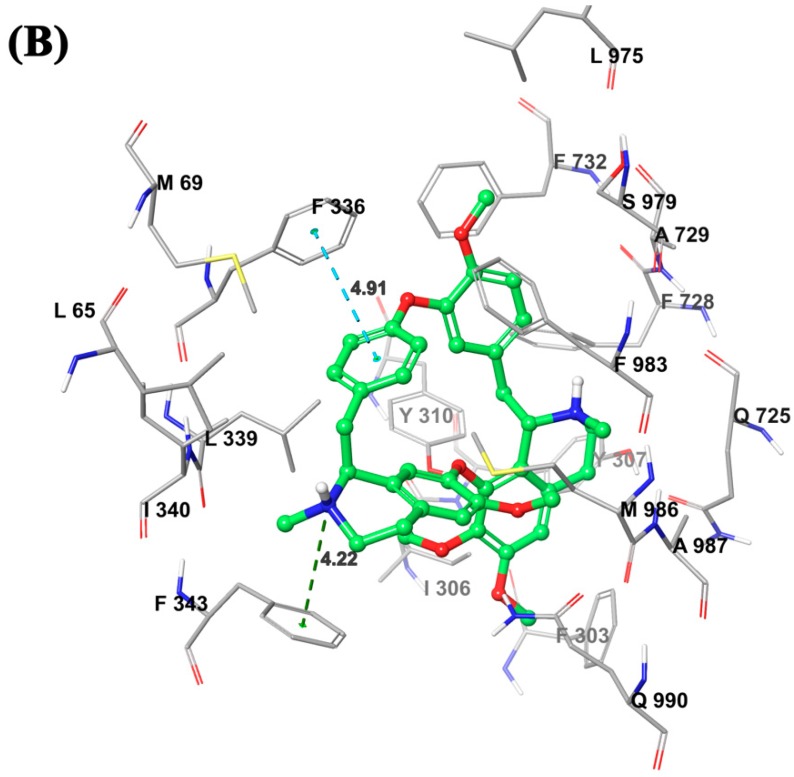
(**A**) Comparison between binding positions of tetrandrine (blue) and verapamil (red) as ball-and-stick models. The human homology ABCB1 is depicted as yellow ribbons. (**B**) Induced-fit docking (IFD) predicted docked conformation of tetrandrine as ball and stick model is shown within the drug-binding site of ABCB1, with the atoms colored as carbon–green, hydrogen–white, oxygen–red, nitrogen–blue. Important amino acids are depicted as sticks with the same color scheme as above except that carbon atoms are represented in grey. Only polar hydrogens were shown. Dotted blue line indicates π-π stacking interaction, while dotted dark green line indicates cation-π interaction. Values of the relevant distances are given in Å.

In order to evaluate the interaction of tetrandrine and ABCB1, the best-scored tetrandrine docking pose within homology-modeled human ABCB1 predicted by IFD simulation was performed and the results was presented in Figure 8. The docked location of tetrandrine in homology human ABCB1 is closed to that of positive inhibitor verapamil (Figure 8A). The docking score of tetrandrine in homology human ABCB1 was −9.016 kcal/mol, indicating a good binding affinity, though not as good as verapamil (docking score −13.715 kcal/mol). The core structure of tetrandrine was stabilized into a hydrophobic cavity which is surrounded by residues L65, M69, I306, F303, Y307, F336, Y310, L339, F343, I340, F728, A729, F732, L975, F983, M986, and A987. The 2′-ionized methylamine group of tetrandrine was involved in cation-π interaction with the F343 phenyl ring. In addition, the phenyl group adjacent to 15′-C atom of tetrandrine was predicted to form interaction of π-π stacking with the phenyl ring of F336.

## 3. Discussion

A wide range of anticancer drugs failed for chemotherapy because of MDR. One of the major reasons of MDR is the overexpression of various membrane proteins called ABC transporters. The most common ABC transporters include multidrug resistance-associated protein-1 (MRP1/ABCC1), P-glycoprotein (P-gp/ABCB1/MDR1), and the breast cancer resistance protein (BCRP/ABCG2). These transporters enhance the excretion of endogenous and exogenous substances including anticancer drugs [29]. Previous studies have demonstrated that agents named chemosensitizers could overcome this phenomenon [30]. Three generations of chemosensitizers have been developed so far. The first-generation reversal agents such as calcium channel blocker verapamil were discovered by Tsuruo [31]. In addition, other drugs such as quinine, quinidine [32], calmodulin inhibitors phenothiazines [30], tamoxifen, and toremifene [33], cyclosporine [34], and the cephalosporins ceftriaxone and cefoperazone [35], were usually used for treating other diseases and were found to bear ABCB1 reversal activity [36]. However, these drugs showed high toxicity and side effects [37]. The second-generation inhibitors such as dex-verapamil, Biricodar (VX710), and valspodar (PSC833) are derivatives from and better than the first-generation agents [38]. The third-generation inhibitors such as Tariquidar (XR9576) [39], Zosuquidar (LY335979) [40], Laniquidar (R101933) [41], and Elacridar (GF120918/GG918) [42] are synthetic small molecules that could interact with ABC transporters [43]. Some of them showed a strong ability to reverse MDR with a dose at the micro- and nаno-molar grades and low toxicity and less side effect. However, their pharmacokinetics is unpredictable [44]. Some clinical trials have shown that these chemosensitizers increased the toxicity of anticancer drugs, and have limited or no benefits for cancer patients [45]. Currently, natural products are expected to lead the fourth generation of ABC inhibitors due to their biodiversity, multiple targets, less side effects, and fewer adverse reactions [46].

In the current study, we explore the potential of using tetrandrine as a chemosensitizer. Verapamil was used as a positive control reversal compound. Cisplatin, which is not a substrate of ABC transporters, was taken as a negative control compound. The MTT assay showed that the IC_50_ values in parental and resistant cells pretreated with tetrandrine alone were almost equal, suggesting that tetrandrine possesses similar cytotoxicity toward both sensitive and resistant cells. This result was consistent with the study of Tian H [47]. Reversal MTT assays also showed that the non-toxic concentration of tetrandrine did not change the IC_50_ values of parental cells KB-3-1, SW620, and HEK293/pcDNA3.1, but diminished the IC_50_ values in a dose-dependence pattern in drug resistant cells SW620/Ad300, KBC2, and HEK293/ABCB1 that overexpressed ABCB1 when used in combination with other chemical drugs such as Doxorubicin, Vincristine, and Paclitaxel. These results suggested that tetrandrine was a potential reversal agent of ABCB1 transporter. We observed that the reversal effect of tetrandrine for vincristine resistance in SW620/Ad300 cell line was much weaker than that in other drug resistant cell lines. This might be due to multiple resistant factors existing in the SW620/Ad300 cell line, although P-gp overexpression is one of the major factors. Further evidence was given by experiments on intracellular accumulation and efflux of [^3^H]-paclitaxel. This showed that, after pretreatment with [^3^H]-paclitaxel and tetrandrine for 1 h, there was significant difference in the intracellular radio of [^3^H]-paclitaxel between tetrandrine or verapamil treatment group and control group, suggesting that tetrandrine and verapamil could maintain intracellular accumulation of [^3^H]-paclitaxel. After 30, 60, and 120 min, intracellular preservation of [^3^H]-paclitaxel was still maintained at a high level, indicating that tetrandrine was able to increase the accumulation of [^3^H]-paclitaxel in ABCB1 overexpressed cells by inhibiting the efflux function of ABCB1. To further understand the reversal mechanism of tetrandrine, we tested the expression of ABCB1 protein and *MDR1* mRNA in SW620/Ad300 and KB-C2 cells after pretreatment with tetrandrine 1.0 µM for 0, 24, 48, and 72 h by using immunofluorescent staining, Western blotting, and real-time PCR methods. We found that tetrandrine increased the sensitivity of anticancer drugs by downregulating the protein expression of ABCB1, but had no effect on the transcriptional level of *MDR1* mRNA, suggesting that the function of tetrandrine in inhibiting the expression of ABCB1 is probably due to post-transcriptional regulation. This result was supported by research on doxorubicin-induced MCF-7/Dox cells [23], additional research from Xu JY et al., [48] who used a KBV200 cell line and tested the expression of *MDR1* mRNA, and by Sun YF. et al., [26], who used MDR Caco-2 and CEM/ADR5000 cancer cells and tested the expression of ABCB1. However, there were different opinions from Shen H. [25] and Chen HY. [19] They used doxorubicin-induced K562 cells and tamoxifen-induced MCF-7/TAM cells, and their results suggested that tetrandrine inhibited the expression of ABCB1 protein and *MDR1* mRNA. There was also a contradicting view from Liming Chen et al. [20], who considered that tetrandrine could increase the intracellular concentration of anticancer drugs but did not reduce the expression of ABCB1 in KBv200 cells by using flow cytometry and Western blotting assay.

As a transporter with ATPase activity, the status of ATPase activity is an important indicator that represents the efficacy of a substrate efflux function. Inhibition of ATPase activity has become one of the mechanisms for reversing MDR by using some chemosensitizers. In this study, ATPase activity of ABCB1 was stimulated in a concentration-dependent manner after treatment with tetrandrine. Therefore, the mechanism of tetrandrine in reversing MDR is not related to the inhibition of ATPase activity, but rather competitively inhibits the function of ABCB1. Docking analysis was conducted to understand the interactions between tetrandrine and ABCB1 transporter. It was predicted that the core structure of tetrandrine was mainly stabilized into a hydrophobic drug-binding cavity of the ABCB1 transporter, with π-π stacking and cation-π interactions formed between tetrandrine and ABCB1. This result indicated that tetrandrine might be a weak substrate of ABCB1, which supports its stimulating effect of ABCB1 ATPase activity. Although the predicted binding affinity of tetrandrine towards ABCB1 was lower than that of verapamil, tetrandrine exhibited comparable performance with verapamil in reversing ABCB1-induced MDR in vitro. On one hand, computational docking analysis using homology modeled human ABCB1 can only be used for prediction of binding affinities with a lot of unknown hits. On the other hand, as functional inhibition and down-regulated protein expression of ABCB1 were both observed, the possibility could not be eliminated that tetrandrine may have multiple mechanisms of action to reverse ABCB1-induced MDR besides of binding to ABCB1. Further study is needed to elucidate possible mechanisms of ABCB1 down regulation by using tetrandrine. Overall, these results provide a clue for designing structure of tetrandrine analogs and using them as ABCB1-mediated MDR reversal agents.

## 4. Materials and Methods

### 4.1. Reagents

Tetrandrine with over 98% purity was purchased from Shanghai Yuanye Biotechnology Company (Shanghai, China), while monoclonal anti-P-glycoprotein antibody, doxorubicin, paclitaxel, vincristine, cisplatin, verapamil, MTT [3-(4,5-dimethylthiazol-yl)-2,5-diphenyl-tetrazolium bromide], ATPase assay kit, ABCB1 antibody (catalogue number p7965) and other chemicals were purchased from Sigma (St. Louis, MO, USA). Alexa flour 488-conjugated goat anti-mouse IgG was purchased from Molecular Probes (Eugene, OR, USA) and [^3^H]-paclitaxel (15 Ci/mmol, MT552) was purchased from Moravek Biochemicals (Brea, CA, USA). Mammalian Protease Inhibitor Cocktail 100X (AMRESCO, LLC), HRP-labeled rabbit anti-mouse secondary IgG antibody was purchased from Santa Cruz (Dallas, TX, USA), and BD Pharmingen^TM^ PI/RNase Staining Buffer were purchased from BD Biosciences (Franklin lake, NJ, USA). Monoclonal anti-GAPDH antibody, Reverse Transcriptase reagent for RT-PCR SuperScript^®^ II, TRIzol^®^ Reagent, Pierce^TM^ BCA protein Assay Reagent A/B, and Pierce^TM^ ECL Western blotting substrate were purchased from Thermo Fisher (Rockford, IL, USA), while sodium orthovanadate was purchased from Alfa Aesar (Ward Hill, MA, USA). Adenosine triphosphate (ATP) was purchased from Amresco (Solon, OH, USA).

### 4.2. Cells Culture

The human epidermoid carcinoma cell KB-3-1 and its resistant cell KB-C2 were used. KB-C2 was generated by treating KB-3-1 cells with increasing concentrations of colchicine. The cell lines HEK293/ABCB1 and HEK293/pcDNA3.1 were generated by transfecting ABCB1 expression vectors and empty vector pcDNA3.1 into the HEK293 cells [49]. The human colon cancer SW620 and its resistant cell SW620/Ad300 cells were generated by selecting increasing concentrations of doxorubicin. All the drug resistant cells overexpressed ABCB1. All cells were cultured in DMEM supplemented with 10% fetal bovine serum and 1% penicillin/streptomycin in an incubator with 5% CO_2_ at 37 °C.

### 4.3. MTT Assay

MTT colorimetric assay was used in this study according to previous descriptions. To evaluate cytotoxicity, parental and resistant cell lines (SW620 and SW620/Ad300, KB-3-1 and KB-C2, HEK293/pcDNA3.1 and HEK293/ABCB1) were seeded into 96-well plate (with 160 μL medium and 5000–6000 cells/well), then gradient concentrations of tetrandrine were added into parallel wells. For the reversal assay, after pretreatment for 1 h with different nontoxic concentrations of tetrandrine (1 μM or 3 μM), and verapamil (3 μM) which was used as the ABCB1 positive control inhibitor, gradient concentrations of each anticancer drugs (20 μL) were added into appropriate wells, and the plates were kept in an incubator for 72 h. After 20 µL of MTT solution (4 mg/mL) was added into wells and the plates were maintained at 37 °C for 4 h, the MTT/medium of each well was discarded carefully and 100 µL DMSO was added. After a short swinging, the light absorbance was detected at 570 nm by accuSkan GO microplate spectrophotometer (Fisher Scientific, Waltham, MA, USA). All of these experiments were repeated three times. IC50 values were calculated according to the formula of the trend line which consisted of at least three effective points.

### 4.4. The Efflux and Accumulation Assay for [^3^H]-Paclitaxel

The drug reversal effect of ABCB1 transporter was examined by evaluating the efflux and accumulation of [^3^H]-Paclitaxel in KB-C2 and KB-3-1 cells. In this experiment, cells were trypsinized and suspended in DMEM medium, and then divided equally into four centrifuge tubes. After incubating with or without tetrandrine or verapamil at 37 °C for 1 h, [^3^H]-Paclitaxel was added into medium and the terminal concentration of [^3^H]-Paclitaxel was 0.1 μM. After being retained for another 1 h at 37 °C, cells were collected and washed with cold PBS. In the accumulation assay, cells were lysed by the lysis solution (0.2% SDS and 1% Triton X-100, pH 7.4) and moved into a scintillation vial. In the efflux assay, cells were resuspended in the fresh medium with or without tetrandrine or verapamil. At 0, 30, 60, and 120 min time points, cells of the same volume were harvested and washed with cold PBS and added with scintillation fluid. Tri-Carb 3110TR Liquid Scintillation Analyzer (PerkinElmer) was used to measure radioactivity.

### 4.5. Extraction of Total Cell Protein and Western Blotting Analysis

KB-C2, KB-3-1, SW620/Ad300, and SW620 cells were cultured in flask with tetrandrine compound (1 μM) for different times: 0, 24, 48, and 72 h. After that, the media were removed and the cells were washed with cold PBS. The lysis buffer 200–300 μL (1 mM EDTA, 150 mM NaCl, 0.1% SDS, 10 mM Tris HCI, Triton X-100, pH 7.5, mixed with 1% Protease inhibitors) was added into the flask. After the supernatant was separated from cells lysis liquid by centrifuging at 12,000 rpm for 30 min at 4 °C, it was stored at −80 °C until it was used. Various proteins in cell lysate were isolated from each other on SDS-PAGE gel according to their molecular weight under electric current action and then transferred onto polyvinylidene fluoride (PVDF) membranes pretreated with formaldehyde. After that, PVDF membrane was blocked with 5% skim milk for 1 h to eliminate nonspecific background, and incubated in a TBST buffer (150 mM NaCl, 0.1% Tween 20, 10 mM Tris HCI, pH 8.0) with a primary monoclonal antibody of ABCB1 (1:500 dilution) or GAPDH (1:1000 dilution) at 4 °C overnight. Subsequently, PVDF membrane was incubated with Goat anti-mouse horseradish peroxide (HRP)-conjugated secondary antibody (1:1000 dilution) for 2 h at room temperature, and luminescent substrate. Strips were exposed on film.

### 4.6. Analysis of ABCB1 ATPase Activity

The activity of ABCB1 ATPase was examined in cell membranes treated with tetrandrine at the concentrations of 0 to 40 µM using the PREDEASY ATPase Kits based on previous descriptions with a slight modification [50].

### 4.7. Immunofluorescence

SW620/Ad300 and SW620 cells 2 × 10^4^ were seeded into 24-well plate overnight, and incubated in 1 μM tetrandrine medium for 0, 24, 48, and 72 h. After washing with 1×PBS twice, the cells were fixed in 4% paraformaldehyde, then treated with 0.25% Triton to increase the permeability of cell membrane. The cells were blocked with 5% BSA and incubated with monoclonal anti-P-Glycoprotein antibody (1:200) at 37 °C for 1 h, and then treated with an Alexa flour 488-conjugated goat anti-mouse IgG (1:1000) for 2 h at room temperature. The nuclei were counterstained by PI. The images were obtained using a fluorescence microscope (THORLABS).

### 4.8. Detecting the mRNA Expression by RT-PCR

Total RNAs were isolated from SW620, SW620/Ad300, KB-3-1, and KB-C2 cells after pretreatment with tetrandrine for 0, 24, 48, and 72 h by using Trizol^®^ Reagent. Total RNA purity and concentration were calculated according to the absorbance of OD_260_ and OD_280_ detected with the UV T60 spectrophotometer. The first strand cDNA synthesis was performed by using Reverse Transcription Super Script^®^ II kits, and 3 mg total RNA was used as a template. The primer sequence of GAPDH was sense 5′-GAGAAGGCTGGGGCTCATTT-3′ and antisense 5′-AGTGATGGCATG GACTGTGG-3′. The primer sequence of ABCB1 was sense 5′-CACCCGACTTACAGATGATG-3′ and antisense: 5′-GTTGCCA TTGACTGAAAGAA-3′. Real-time PCR was performed with Agilent Technologies Aria Mx Real-Time PCR System. The expression of ABCB1was quantified by the 2^−ΔΔCt^ method [51]. All experiments were performed in triplicate.

### 4.9. Molecular Modeling

The tetrandrine structure was input by the fragment dictionary of Maestro v11.1 and went through energy minimization using OPLS3 force filed by Macromodel v11.5. The structure was prepared by LigPrep v4.1 with all possible tautomers and ring conformations, and different protonation state at pH 7.0 ± 2.0. Both Macromodel v11.5 and LigPrep v4.1 are from Schrödinger, LLC, (New York, NY, USA). The low-energy 3D structure was subjected to conformational search to obtain at most 50 conformers, with a filtering process used to exclude redundant conformers according to the setting of maximum relative energy difference of 5 kcal/mol and maximum atom deviation of 0.5 Å. The output file containing 50 unique ligand conformers was used as input for docking simulation into human homology ABCB1. The human ABCB1 homology model was a kind gift from S. Aller and was generated based on the mouse ABCB1 protein (PDB ID: 4M1M). All identified drug interacting amino acid residues were selected as centroids by the receptor docking grid with a length of 25 Å [52]. The tetrandrine binding with a human ABCB1 homology model in flexible docking simulation was performed using the extra precision (XP) mode of Glide v7.4 (Schrödinger, LLC, New York, NY, USA). The Glide Emodel value was ranked to identify the best docked pose among multiple conformations [53]. To further optimize the simulated binding between the ligand and receptor, the binding position of tetrandrine with the best Glide Emodel value from Glide XP docking process was subjected to induced-fit docking (IFD) using Glide v7.4. The defaulted parameters were used in IFD protocol, and the Glide g score, which indicates the approximate ligand binding free energy [34], was calculated and expressed as kcal/mol.

### 4.10. Statistical Analysis

Statistical analysis was performed using the software SPSS 13.0 for windows (IBM). All experiments were performed in triplicate and repeated at least three times. One-Way ANOVA was used for comparison between groups. The greyscale ratio of Western blotting was calculated using Image J 1.46r. Values were shown as mean ± SD. The significance level was set at *p* < 0.05.

## Figures and Tables

**Table 1:** Cytotoxicity of tetrandrine in parental and drug resistant cancer cells (Mean ± SD).

IC_50_ ± SD (µM) ^a^ [Resistance folds] ^b^					
SW620	SW620/Ad300	KB-3-1	KB-C2	HEK293/pcDNA3.1	HEK293/ABCB1
6.848 ± 0.338	7.094 ± 0.569 [1.0]	14.315 ± 1.676	9.68 ± 0.085 [0.8]	6.65 ± 0.552	7.57 ± 0.339 [1.1]

The cytotoxicity of tetrandrine in several pairs of parental and drug resistant cancer cell lines. ^a^ IC_50_ represent mean ± SD of three independent experiments performed in triplicate. ^b^ Resistance folds (values in square brackets) were calculated by dividing the IC_50_ values of resistant cells by the IC_50_ of parental cells.

**Table 2:** Reversal effect of tetrandrine in three pairs of parental and resistant cell lines (Mean ± SD).

**Treatment**	**IC_50_ ± SD ^a^ (μM, Resistance Fold ^b^)**	
	**SW620**	**SW620/Ad300**
Doxorubicin	0.135 ± 0.066 [1.0]	8.665 ± 0.686 [64.2]
+Tetrandrine 1 μM	0.138 ± 0.078 [1.0]	0.655 ± 0.049 [4.9] ^#^
+Tetrandrine 3 μM	0.119 ± 0.038 [0.9]	0.197 ± 0.002 [1.5] ^#^
+Verapamil 3 μM	0.108 ± 0.014 [0.8]	2.370 ± 0.693 [17.6] ^#^
Vincristine	0.268 ± 0.032 [1.0]	141.060 ± 25.977 [526.3]
+Tetrandrine 1 μM	0.274 ± 0.029 [1.0]	98.797 ± 25.025 [368.6] *
+Tetrandrine 3 μM	0.348 ± 0.039 [1.3]	38.710 ± 8.976 [144.4] ^#^
+Verapamil 3 μM	0.360 ± 0.015 [1.3]	42.144 ± 2.625 [157.2] ^#^
Paclitaxel	0.019 ± 0.001 [1.0]	108.990 ± 5.996 [5736.3]
+Tetrandrine 1 μM	0.015 ± 0.002 [0.8]	6.030 ± 0.749 [317.4] ^#^
+Tetrandrine 3 μM	0.018 ± 0.001 [1.0]	0.373 ± 0.047 [19.6] ^#^
+Verapamil 3 μM	0.024 ± 0.001 [1.3]	4.790 ± 0.509 [252.1] ^#^
Cisplatin	2.245 ± 0.869 [1.0]	2.354 ± 0.558 [1.1]
+Tetrandrine 1 μM	2.614 ± 0.361 [1.2]	2.701 ± 1.563 [1.2]
+Tetrandrine 3 μM	2.882 ± 0.556 [1.3]	2.198 ± 1.115 [1.0]
+Verapamil 3 μM	2.925 ± 0.728 [1.3]	2.512 ± 0.247 [1.1]
**Treatment**	**IC_50_ ± SD ^a^ (μM, Resistance Fold ^b^)**	
	**KB-3-1**	**KB-C2**
Doxorubicin	0.573 ± 0.137 [1.0]	14.115 ± 3.854 [24.6]
+Tetrandrine 1 μM	0.545 ± 0.035 [1.0]	0.319 ± 0.057 [0.6] ^#^
+Tetrandrine 3 μM	0.470 ± 0.014 [0.8]	0.277 ± 0.008 [0.5] ^#^
+Verapamil 3 μM	0.585 ± 0.007 [1.0]	0.520 ± 0.028 [0.9] ^#^
Vincristine	0.068 ± 0.001 [1.0]	22.430 ± 4.059 [329.9]
+Tetrandrine 1 μM	0.071 ± 0.014 [1.0]	0.258 ± 0.002 [3.8] ^#^
+Tetrandrine 3 μM	0.060 ± 0.003 [0.9]	0.015 ± 0.001 [0.2] ^#^
+Verapamil 3 μM	0.066 ± 0.002 [1.0]	0.056 ± 0.007 [0.8] ^#^
Paclitaxel	0.029 ± 0.005 [1.0]	13.070 ± 0.203 [450.7]
+Tetrandrine 1 μM	0.033 ± 0.009 [1.1]	0.231 ± 0.014 [7.9] ^#^
+Tetrandrine 3 μM	0.031 ± 0.004 [1.1]	0.083 ± 0.002 [2.9] ^#^
+Verapamil 3 μM	0.027 ± 0.006 [0.9]	0.422 ± 0.071 [14.6] ^#^
Cisplatin	5.995 ± 0.262 [1.0]	4.615 ± 0.092 [0.8]
+Tetrandrine 1 μM	4.925 ± 0.247 [0.8]	4.540 ± 0.382 [0.8]
+Tetrandrine 3 μM	4.905 ± 0.318 [0.8]	4.620 ± 0.141 [0.8]
+Verapamil 3 μM	5.890 ± 0.169 [1.0]	4.410 ± 0.127 [0.7]
**Treatment**	**IC_50_ ± SD ^a^ (μM, Resistance Fold ^b^)**	
	**HEK293/pcDNA3.1**	**HEK293/ABCB1**
Doxorubicin	0.072 ± 0.024 [1.0]	0.829 ± 0.060 [11.5]
+Tetrandrine 1 μM	0.051 ± 0.001 [0.7]	0.056 ± 0.012 [0.8] ^#^
+Tetrandrine 3 μM	0.041 ± 0.014 [0.6]	0.039 ± 0.006 [0.5] ^#^
+Verapamil 3 μM	0.046 ± 0.004 [0.6]	0.177 ± 0.166 [2.5] ^#^
Vincristine	0.635 ± 0.049 [1.0]	6.797 ± 2.216 [10.7]
+Tetrandrine 1 μM	0.530 ± 0.014 [0.8]	0.865 ± 0.035 [1.4] ^#^
+Tetrandrine 3 μM	0.478 ± 0.025 [0.8]	0.621 ± 0.011 [1.0] ^#^
+Verapamil 3 μM	0.618 ± 0.060 [1.0]	0.737 ± 0.019 [1.2] ^#^
Paclitaxel	1.825 ± 0.007 [1.0]	23.425 ± 0.071 [13.0]
+Tetrandrine 1 μM	2.095 ± 0.106 [1.2]	4.930 ± 0.207 [2.0] ^#^
+Tetrandrine 3 μM	1.950 ± 0.127 [1.1]	0.880 ± 0.029 [0.5] ^#^
+Verapamil 3 μM	2.380 ± 0.099 [1.3]	1.833 ± 0.042 [1.0] ^#^
Cisplatin	2.240 ± 0.212 [1.0]	2.067 ± 0.402 [0.9]
+Tetrandrine 1 μM	2.555 ± 0.304 [1.1]	1.790 ± 0.192 [0.8]
+Tetrandrine 3 μM	2.735 ± 0.502 [1.2]	1.667 ± 0.053 [0.7]
+Verapamil 3 μM	2.480 ± 0.325 [1.1]	1.958 ± 0.094 [0.9]

MTT assay: tetrandrine reverses the ABCB1-mediated drug resistance in ABCB1 overexpressing cell lines. ^a^ IC_50_ values represent mean ± SD of three independent experiments performed in triplicate. ^b^ Resistance fold (values in square brackets) was calculated by dividing the IC_50_ values of substrates in the presence or absence of inhibitors by the IC_50_ of parental cells without an inhibitor. * *p* < 0.05, ^#^ *p* < 0.01 versus the no inhibitor group.
